# Supercritical Fluid Extraction to Valorise Fruit and Vegetable by-Products

**DOI:** 10.3390/foods15101692

**Published:** 2026-05-12

**Authors:** Miriana De Feo, Amalia Conte, Matteo Alessandro Del Nobile

**Affiliations:** 1 Department of Humanistic Studies, Letters, Cultural Heritage, Educational Sciences, University of Foggia, Via Arpi, 71121 Foggia, Italy; miriana.defeo@unifg.it; 2 Department of Economics, Management and Territory, University of Foggia, Via A. da Zara, 71122 Foggia, Italy; matteo.delnobile@unifg.it

**Keywords:** supercritical fluid extraction (SFE), green chemistry, agro-industrial by-products, bioactive compounds, circular bioeconomy

## Abstract

The global transition toward a circular bioeconomy requires the urgent valorization of agro-industrial by-products, specifically fruit and vegetable residues, which represent a significant environmental burden but also a rich source of high-value bioactive compounds. This review critically examines Supercritical Fluid Extraction (SFE) using carbon dioxide (CO_2_) as a highly efficient and versatile technological platform for the sustainable recovery of specialty lipids and secondary metabolites. By analysing the thermodynamic foundations of the supercritical state, the study explores how the tuneable density and transport properties of supercritical CO_2_ (SC-CO_2_) facilitate molecular preservation compared to conventional solvent-based methods. The discussion focuses on the mechanistic aspects of the process, including the role of green co-solvents in overcoming polarity barriers and the application of kinetic models to describe mass transfer phenomena in complex matrices. Unlike general surveys, this work integrates technical-economic considerations with critical analysis of SFE’s role within a multi-stage biorefinery framework, essential for aligning economic profitability with the ecological mandate of strategic molecular recovery.

## 1. Introduction

The extraction of bioactive compounds from natural sources is currently facing a transformative period driven by a dual necessity: the demand for high-quality functional ingredients and the mandatory transition toward sustainable industrial practices. For decades, the food industry has utilized conventional solvent extraction as the primary tool for recovering secondary metabolites. However, these traditional methods are increasingly criticized due to their environmental and toxicological footprints. Within the European regulatory framework, specifically under the REACH legislation, many petrochemical solvents are being restricted or phased out. This shift is primarily due to their volatility, flammability, and the risk of leaving toxic residues in final food products, which contradicts the modern “Green Chemistry” paradigm [1]. The transition toward a circular economy requires the implementation of green chemistry principles to meet sustainable development goals. This involves moving away from hazardous solvents and energy-intensive processes toward “intensified” techniques that prioritize both environmental safety and process efficiency [2,3]. Conventional methods like Soxhlet extraction or maceration, while simple, often require large volumes of organic solvents and long processing times, which are no longer sustainable in a market that demands “clean-label” products [4].

A critical limitation of classical extraction is the inherent instability of the target molecules. Plant-derived bioactives, such as phenolic compounds and carotenoids, are essential for their antioxidant activities [5]. However, these compounds are highly sensitive to the conditions typically found in conventional extraction, such as prolonged exposure to heat, light, and atmospheric oxygen [6]. For instance, carotenoids possess a system of conjugated double bonds that makes them extremely vulnerable to thermal oxidation and isomerization [7]. When these compounds are extracted using boiling solvents, their biological potency is often significantly reduced. Similarly, polyphenols can undergo oxidative degradation or polymerization when handled in the presence of air at elevated temperatures [8]. This molecular fragility necessitates the use of technologies that can isolate these compounds without altering their native chemical structure. Emerging green techniques are being explored to ensure that the final food products retain functional appearance and health benefits [9,10].

Supercritical Fluid Extraction (SFE), particularly employing carbon dioxide (CO_2_), represents a definitive solution to these challenges, positioning itself as a “Green Preservation” technology. Supercritical CO_2_ reaches its critical state at relatively low temperatures and pressures, which is ideal for the recovery of thermolabile compounds [11]. Its gas-like diffusivity and liquid-like density allow it to penetrate deeply into complex plant matrices, ensuring high mass transfer rates without the need for high temperatures [12]. The fundamental advantage of SFE lies in the inert, oxygen-free, and light-protected environment within the extractor, shielding sensitive antioxidants from the degradative pathways [13]. Furthermore, SFE offers an unparalleled degree of selectivity. By adjusting the pressure and temperature parameters, the solvation power of the fluid can be precisely tuned to target specific molecules. However, it should be noted that this selectivity is strictly dependent on the operating conditions; for instance, at pressures exceeding 200–250 bar, the increased density of SC-CO_2_ may lead to the co-extraction of non-polar matrix components such as waxes and fats [14]. Similarly, the potential co-extraction of heavy metals must be considered if they are present in organometallic forms. This adjustability is often enhanced by coupling SFE with advanced analytical techniques or chromatography for the precise determination of compounds like carotenoids in dietary supplements [15,16]. Beyond simple extraction, SFE is also used in the formulation of bio-stimulants and cosmetics, preserving the bioactivity of algal and plant extracts [17].

The commercial adoption of SFE is often analysed through the balance between initial investment and long-term operational costs. It is undeniable that SFE systems require a higher capital expenditure compared to atmospheric extraction vats, due to the necessity for high-pressure stainless-steel vessels [18]. Industrial-scale SFE systems typically involve a capital expenditure (CAPEX), depending on vessel capacity and automation level. However, the operational cost (OPEX) at an industrial scale is remarkably competitive, as the solvent is recovered and recycled through simple phase modulation rather than energy-intensive distillation. The economic sustainability of the process is deeply rooted in its thermodynamic efficiency, which can be precisely modelled and visualized through the Mollier diagram (enthalpy vs. pressure). The Mollier diagram is fundamental for SFE as it maps the energy cycle of CO_2_, illustrating how the fluid transitions from a gas or liquid to a supercritical state and back again [19]. Unlike conventional methods, where the latent heat of vaporization for organic solvents represents a massive energy drain, the SFE cycle operates by modulating pressure and temperature to trigger precipitation. By following the paths defined in the Mollier diagram, industrial plants can optimize the recycling of CO_2_ with minimal energy expenditure. This efficient phase management significantly lowers the operating expenditures, as the solvent is recovered by simple depressurization rather than energy-intensive distillation [11,13]. Beyond thermodynamic efficiency, the industrial scale-up of SFE for biomass valorization must address specific logistical and operational challenges. The high capital expenditure (CAPEX) for high-pressure equipment is balanced by low solvent costs, provided the efficient CO_2_ recycling and energy-integrated systems are employed to minimize electricity demand. However, the economic viability is also sensitive to feedstock logistics; the seasonality of fruit and vegetable residues requires strategic planning to ensure a consistent annual throughput. Furthermore, the market value of the recovered compounds must justify the processing costs, often necessitating a “zero-waste” approach where multiple fractions (e.g., oils, pigments and fibers) are commercialized to offset the initial investment. From an environmental perspective, the sustainability of SFE is quantified by its low energy demand for solvent recovery and high CO_2_ recycling rates (often >95%), which significantly reduce the carbon footprint compared to energy-intensive distillation of organic solvents. When coupled with renewable energy sources and the full valorization of the exhausted biomass, SFE aligns, with the quantitative requirements of Life Cycle Assessment (LCA) for green biorefineries. Consequently, SFE yields a solvent-free extract immediately ready for food-grade applications, justifying the initial capital through the production of premium, high-value ingredients that meet the strictest safety standards [4].

While several reviews have documented the use of SC-CO_2_ for extracting plant-derived compounds, the present work offers a distinct contribution by bridging the gap between fundamental thermodynamic modelling and industrial-scale biorefinery integration. Specifically, this review moves beyond a simple cataloguing of extraction yields to critically evaluate how kinetic models, such as the “broken and intact cell” model, can be strategically used to optimize the sequential cascade valorization of fruit and vegetable by-products. By integrating an analysis of operational costs (CAPEX/OPEX) and the practical limitation of co-solvent and NADES usage at scale, this study provides a comprehensive roadmap for transitioning from laboratory-scale experiments to viable industrial “clean-label” production.

## 2. Methods

The present review was conducted through a systematic literature search across major scientific databases, including Scopus, Web of Science, and Google Scholar. The survey was primarily restricted to peer-reviewed publications from the last decade to ensure the inclusion of the most recent technological advancements and sustainability analyses. The search strategy employed specific keywords such as “supercritical fluid extraction,” “SC-CO_2_,” and “green solvents,” which were cross-referenced using Boolean operators with terms related to a wide range of biological matrices. Specifically, the search encompassed both fruit-processing by-products (pomace, citrus peels, fruit seeds) and vegetable residues (vegetable waste, leafy by-products, root-derived residues). Inclusion criteria prioritized studies providing quantitative data on thermodynamic modelling, extraction kinetics, and environmental impact assessments. This methodological approach allowed for a rigorous synthesis of the state-of-the-art regarding supercritical technology within the framework of the circular bioeconomy.

## 3. SFE: Molecular Preservation and Advanced Biorefinery Integration

### 3.1. SFE Mechanisms

The technological superiority of SFE over conventional liquid-solvent methods is fundamentally rooted in the unique, tuneable thermodynamics of fluids beyond their critical point. As rigorously discussed in the seminal work by Brunner [20], CO_2_ reaches its supercritical state (SC-CO_2_) when both temperature and pressure exceed 31.1 °C and 7.38 MPa, respectively. In this regime, the fluid exists in a state of physicochemical hybridity: it possesses gas-like transport properties, such as high diffusivity and low viscosity, while simultaneously maintaining a liquid-like solvating power that is directly proportional to its density [21]. This dual nature is the mechanical engine behind its efficiency. In a standard solid–liquid extraction (SLE), the solvent’s ability to penetrate the internal capillaries of a matrix is limited by surface tension and slow molecular diffusion. Conversely, SC-CO_2_ exhibits near-zero surface tension, allowing it to bypass macro-pores and infiltrate the microscopic cellular architecture. This leads to a significant reduction in the internal mass transfer resistance, a phenomenon that Dmitrienko et al. [22] identify as the “kinetic advantage” of supercritical systems.

From a molecular preservation standpoint, the most critical factor remains the low operating temperature. Many bioactive metabolites, particularly carotenoids and polyunsaturated fatty acids (PUFAs), are governed by delicate conjugated double-bond systems that are highly susceptible to thermal degradation and *trans-cis* isomerization [23]. By maintaining the process within a narrow thermal window of 35–45 °C, SFE ensures that the extracted solutes retain their native biological configuration, a feat nearly impossible with Soxhlet or reflux-based methods [24]. The industrial appeal of this technology is further underscored by its scalability and environmental footprint. Knez et al. [24] highlight how the low-energy recovery of the solvent via depressurization reduces the carbon footprint and operational costs in large-scale applications. From an analytical perspective, SFE represents the most advanced method for the extraction of organic compounds from complex solid samples, effectively replacing archaic techniques with more selective and rapid protocols [23]. Beyond thermal management, the mechanical superiority of SFE is exemplified by its ability to provide a strictly anaerobic extraction environment. Traditional extraction processes often expose sensitive solutes to atmospheric oxygen, which, catalysed by light or residual heat, triggers the formation of reactive oxygen species (ROS). This leads to the irreversible primary and secondary oxidation of phenolic compounds and lipids [25].

SFE operates through a mechanism of induced anoxia. The high-pressure flow of CO_2_ acts as a physical displacement agent, purging all interstitial air from the extraction vessel. This creates an inert micro-environment that prevents the oxidation of labile molecules, such as tocopherols or essential oils, at the very moment of their release from the matrix [26]. Recent longitudinal studies on vegetable oils and tocopherols demonstrate that SC-CO_2_ extracts possess a significantly higher induction period and lower peroxide values compared to hexane-extracted counterparts [27]. This mechanical shielding is essential for producing high-purity extracts destined for the pharmaceutical and nutraceutical markets, where molecular integrity is a non-negotiable quality parameter.

The non-polar nature of SC-CO_2_, while ideal for lipids, has historically been a barrier for the recovery of hydrophilic bioactives. The evolution of “modifier” chemistry has addressed this through the integration of polar entrainers. While ethanol remains the gold standard for green co-solvents [28], it is crucial to note that from a practical industrial perspective, the concentration of such modifiers is typically limited to no more than 2% (*w*/*w*). Exceeding this threshold is often unfeasible at large scales due to increased complexities in CO_2_ recycling and the challenge of maintaining a solvent-free extract. The most significant recent breakthrough is the integration of Natural Deep Eutectic Solvents (NADES) [29]. NADES are not merely additives; they are designer solvents formed by the complexation of primary metabolites, such as choline chloride, sugars, and organic acids, that create a hydrogen-bonded network capable of solubilizing high concentrations of polyphenols [30]. The synergy between SFE and NADES represents the current frontier of green chemistry. However, it must be emphasized that NADES-SFE integration is a field that has only recently begun to be explored. To date, there is no robust evidence to support its implementation at a large industrial scale, and its scalability remains to be proved through further pilot studies. Particularly, the development of switchable solvents allows researchers to modulate the hydrophobicity of the system in real-time by adjusting the CO_2_ pressure [31]. This mechanical tuning of the solvent’s polarity enables the selective extraction of different classes of antioxidants within a single operational cycle, dramatically reducing the environmental footprint of the process. Herrero [30] further notes that this integration allows for the recovery of complex polyphenols that were once considered unextractable by CO_2_ alone, effectively bridging the gap between lipophilic and hydrophilic extraction regimes. The mechanics of preservation and the overall extraction efficiency also depend heavily on the physical state of the biomass before it enters the supercritical loop.

### 3.2. Pre-Treatment

Pre-treatment steps, including drying, milling and moisture control, are decisive factors that dictate the accessibility of target compounds. While Drying (e.g., air-drying or lyophilization) is necessary to prevent water from acting as a barrier to SC-CO_2_ diffusion, the residual moisture content must be strictly controlled; typically, levels between 5% and 12% are ideal to facilitate mass transfer without causing vessel clogging. Barba et al. [32] demonstrate that pre-treatments such as lyophilization are superior to conventional drying because they maintain the microporous structure of the tissue, preventing the collapse of cellular capillaries. Furthermore, particle-size reduction through milling increases the specific surface area, shortening the diffusion path fort the supercritical fluid. However, over-milling must be avoided to prevent channelling effects or the excessive release of waxes. These physical modifications, combined with mechanical intensifiers such as Ultrasound-Assisted Extraction (UAE) introduces a disruptive force through acoustic cavitation. The application of ultrasonic waves, a hybrid method known as UASFE, induces a sono-poration effect. As explored by Nam et al. [33], micro-bubbles implode near the cell surface, creating localized micro-jets that fracture the cell wall barriers and ensuring that even resilient lignocellulosic tissue can be effectively valorised. This mechanical disruption facilitates a faster equilibrium between the matrix and the solvent phase, allowing for higher yields at lower pressures. This is crucial for unconventional matrices, such as edible insects or resilient lignocellulosic tissues, where volatile profiles and quality characteristics are paramount [33]. By reducing the residence time in the extractor, UASFE further minimizes the risk of molecular degradation, even under supercritical conditions.

### 3.3. SFE Phases

To understand why SFE “works better,” one must examine the internal kinetics of the process. The “Broken and Intact Cell” model proposed by Sovová and Stateva [21] provides the mathematical backbone for this understanding. The extraction is typically divided into two distinct phases:

1. The CER (Constant Extraction Rate) Period: where the solute on the surface of the broken cells is easily accessible.
2. The DC (Falling Rate) Period: where diffusion from the intact inner cells becomes the limiting factor.

The mechanical advantage of SFE lies in its ability to shorten the DC period by utilizing the high diffusivity of SC-CO_2_. By manipulating the flow rate and pressure, operators can optimize the transition between these phases, a level of control that is significantly more difficult to achieve in stagnant liquid extractions. This kinetic precision is what allows for the high purity reported in tocopherol recovery from vegetable oils [27] and the enzymatic-inhibitory effects found in rosemary by-products [25].

The pinnacle of SFE mechanics is its application within a Sequential Cascade Valorization (SCV) framework. This approach moves beyond the concept of extract and discard, treating biomass as a refinery feedstock where components are separated based on their solubility gradients. King and Srinivas [34] were among the first to propose a multi-unit processing approach using sub- and supercritical fluids as a logical separation scheme.

In a typical cascade setup, the fractionation proceeds as follows:

- Stage I (Lipophilic Fractionation): Pure SC-CO_2_ at moderate pressures (10–20 MPa) is employed to isolate essential oils, terpenes, and non-polar lipids. This has been successfully applied to *Agaricus brasiliensis* mushrooms [35] and grape seeds [36], where the lipophilic fraction is recovered without polar contamination.
- Stage II (Polar Bioactive Recovery): High-pressure SC-CO_2_ (30–50 MPa) combined with a polar modifier (Ethanol/NADES) or an intermediate switch to subcritical water (SW) targets the phenolic fraction. Xynos et al. [37] demonstrated this by producing extracts enriched in oleuropein from olive leaves. Similarly, Hayrapetyan et al. [38] and Da Porto et al. [28] utilized water and ethanol co-solvents to recover proanthocyanidins and other high-value molecules from grape pomace, proving that sequential extraction significantly increases the total antioxidant yield.
- Stage III (Structural Upcycling): The exhausted solid residue, now free of waxes and inhibitory phenolics, serves as a high-purity substrate. Kehili et al. [39] and Niedzwiecki et al. [40] describe how tomato processing by-products can be fractionated into lycopene-rich oils followed by phenolic recovery, leaving a clean residue. This “novel cascade process” ensures that every fraction of the biomass, from oils to fibres, is valorised [40].

The effectiveness of the cascade approach is best understood through comparative results across different matrices. For instance, Mohamed et al. [36] found that grape seed oils extracted by SFE exhibited superior bioactive profiles compared to those obtained via organic solvents, specifically regarding tocopherol content. This aligns with the findings of Mazzutti et al. [35] on *Agaricus brasiliensis*, where SFE parameters were optimized to maximize antimicrobial and antioxidant activities, demonstrating that the “one-size-fits-all” approach of OSE is inferior to the selectivity of SFE. Furthermore, the work of Kehili et al. [39] on Tunisian tomato by-products highlights the economic feasibility of biorefinery processing. By separating the skin from the seeds and applying sequential extraction, they created added value from materials previously considered environmental waste. This is echoed by Niedzwiecki et al. [40], who emphasize that using CO_2_ and water as sequential solvents represents the ultimate “green” separation strategy for the food processing industry.

## 4. The Dynamics of SFE for Bioactive Recovery from Vegetable Residues

### 4.1. Leaf-Based by-Products

The transition toward a global circular bioeconomy necessitates a fundamental redefinition of agricultural by-products, moving away from the traditional view of these materials as disposal burdens toward a strategic understanding of them as reservoirs of high-value phytochemicals. Within this framework, SFE has emerged as the definitive technology for the surgical recovery of bioactive compounds from vegetable bio-residues, offering a level of molecular preservation and environmental safety that conventional solid–liquid extraction protocols, often reliant on neurotoxic or flammable solvents like hexane, methanol, and chloroform, simply cannot replicate [41]. The fundamental mechanical advantage of SFE is rooted in the tuneable thermodynamics of carbon dioxide (CO_2_) beyond its critical point, where the fluid exhibits a unique hybrid of gas-like transport properties, characterized by high diffusivity and near-zero surface tension, and liquid-like solvation power that can be modulated by varying the density of the medium. This duality facilitates the deep penetration of SC-CO_2_ into complex, lignin-reinforced plant cell walls, effectively bypassing the mass transfer resistances and stagnant boundary layers that typically limit the efficiency of organic solvents during the leaching process [42,43]. This capability is particularly critical for leaf-based by-products, such as those derived from *Olea europaea* L. and *Cynara cardunculus*, which are rich in thermolabile secoiridoids, chlorophylls, and complex antioxidant networks that are highly sensitive to the thermal and oxidative stresses encountered in traditional Soxhlet or reflux systems [44,45]. As evidenced by the extensive work of Kyriakoudi et al. [46] and Borjan et al. [47], the application of SC-CO_2_ allows for the highly selective fractionation of chlorophyll (a and b) and lipophilic phenols by precisely manipulating fluid density through minute pressure-temperature adjustments. In the specific case of olive leaves, optimal recovery of tocopherols and non-polar lipids is frequently achieved at pressures ranging from 250 to 300 bar while maintaining temperatures strictly below 50 °C to prevent the thermal degradation or polymerization of the bioactive scaffolds [48,49]. Unlike traditional maceration, SFE operates in a strictly anoxic and light-shielded environment, providing an intrinsic protective barrier against the oxidative degradation of delicate pi-bonds in molecules like oleuropein and α-tocopherol. This chemical integrity significantly enhances the biological potency and pharmacological relevance of the resulting extracts, as demonstrated by their superior cytotoxicity and inhibitory effects against human breast cancer cell lines in comparative studies that identify SFE-derived fractions as more balanced in their synergistic phytochemical profiles [50,51]. Furthermore, the logistical complexity of extracting polar metabolites, which are often the primary targets in vegetable waste valorization, has led to the evolution of intensified green protocols. While SC-CO_2_ is inherently non-polar, its solvation window can be significantly expanded through the strategic addition of green modifiers, most notably bio-ethanol, or through the integration of subcritical water in sequential “green” biorefinery steps that utilize the high pressure of the system to maintain water in a liquid state at elevated temperatures, thereby reducing its dielectric constant to match that of organic alcohols [52,53]. These modified systems allow for the recovery of high-purity phenolic concentrates and the production of micronized antioxidant particles via supercritical antisolvent processes, which improve the dispersibility and stability of the final ingredient for the pharmaceutical and nutraceutical sectors [54]. The role of pre-extractive engineering and biomass preparation is a dominant factor in determining industrial feasibility, as the choice of drying method, comparing lyophilization to convective thermal drying, dictates the final microporous architecture of the vegetable tissue, which in turn directly influences the effective diffusion coefficient and the internal mass transfer kinetics during the extraction cycle [55,56]. Modern approaches increasingly utilize mechanical pre-treatments, such as UAE or microwave-assisted extraction (MAE), to induce micro-fractures in the cell wall matrix. These techniques, when coupled with SFE, effectively reduce the induction time and total solvent consumption while maximizing the cumulative yield of targeted secoiridoids like oleuropein from wild olive leaves and other pruning wastes [57,58].

### 4.2. Peels, Seeds and Pomace

When expanding the scope to roots and tubers, the mechanical precision of SFE remains unparalleled. In the processing of carrot (*Daucus carota*) peels and pomace, by-products that constitute nearly 30% of industrial carrot processing waste, the stabilization of carotenoids (β-carotene, α-carotene, and lutein) is achieved through a strictly controlled thermal regime that circumvents the geometric trans-to-cis isomerization often triggered by traditional high-temperature reflux methods [59,60]. Research suggests that a pressure of 300 bar and a temperature of 45 °C represent a thermodynamic “sweet spot” for carotenoid recovery, where the balance between fluid density and solute vapor pressure is optimized to favour high extraction rates without excessive pumping energy costs [61,62]. Similarly, the valorization of beetroot (*Beta vulgaris*) by-products and potato (*Solanum tuberosum*) peels targets the recovery of betalains and chlorogenic acids, respectively. For these matrices, SFE serves as a high-throughput alternative to methanol-based leaching, producing extracts that are immediately suitable for clean-label food applications as natural colorants or shelf-life extenders for fresh-cut produce, effectively reducing the chemical footprint of the food supply chain [63,64,65]. The systemic efficiency of SFE is further highlighted in the processing of turmeric roots (*Curcuma longa*), where the challenge of separating volatile essential oils from non-volatile curcuminoids is addressed through multi-stage pressure reduction that allows for fractional collection. Recent “co-extraction” paradigms have even demonstrated that processing turmeric roots alongside dried coconut shreds allows the natural coconut lipids to act as a synergistic carrier, improving the bio-accessibility and anti-cancer properties of the curcuminoids within the human digestive tract [66,67]. The Brassicaceae family, including broccoli, cauliflower, and kale, presents a unique biochemical challenge due to the presence of glucosinolates and their hydrolysis products, the isothiocyanates. SFE allows for the extraction of sulforaphane and other bio-active isothiocyanates from broccoli by-products at low temperatures, preserving their potent antioxidant and cosmetic-grade properties while minimizing the formation of undesirable off-flavours associated with heat-induced degradation [68,69]. A critical development in this sector is the dual-valorization of broccoli stems and leaves; while the supercritical fluid removes high-value pigments and sulfuric compounds, the remaining solid residue undergoes structural functionalization to improve its dietary fibre profile, effectively transforming a low-value discard into a high-fiber structured ingredient for the bakery or meat-alternative industries [70]. This logic of “zero-waste” is equally applicable to the tomato and pumpkin industries. In tomato processing waste (peels and seeds), SFE is utilized to isolate lycopene and β-carotene, enriched oleoresins at pressures up to 500 bar. These extracts exhibit significantly higher oxidative stability compared to those obtained via petroleum-ether extraction, making them ideal for high-end nutraceutical formulations and dietary supplements [71,72,73,74]. The detailed supercritical fluid extraction conditions for the recovery of bioactive compounds from vegetable matrices and their operational comparison with traditional methods are summarized in Table 1 and Table 2, respectively. Similarly, the recovery of pumpkin seed oil and carotenoids from the peel of *Cucurbita moschata* involves the careful optimization of particle size and flow rates to overcome the intra-particle diffusion limits characteristic of these oil-rich matrices, often achieving yields that surpass traditional cold-pressing methods in terms of antioxidant content and shelf-life stability [75,76,77,78]. The cumulative findings from these diverse vegetable matrices suggest that SFE is not merely a replacement for organic solvents, but the centrepieces of a sophisticated biorefinery cascade. This cascade logic involves the sequential fractionation of biomass: first, the isolation of high-purity lipids and pigments with pure SC-CO_2_, second, the recovery of polyphenols and glycosylated compounds using CO_2_ with polar co-solvents, and finally, the upcycling of the spent lignocellulosic residue into fermentable sugars, animal feed, or renewable energy sources, thereby ensuring that no part of the agricultural stream is lost to the landfill [79]. The analytical strategies deployed for the green characterization of these agri-food bio-residues must therefore evolve to include comprehensive bioactivity evaluations, ensuring that the final extracts meet pharmaceutical-grade standards for bio-availability, chemical stability, and safety [80,81]. As we look toward the 2026–2030 period, it is essential to recognize that SFE is the only green extraction technology that has maintained a successful industrial presence for over three decades in the food, cosmetic, and pharmaceutical sectors. The scalability of SFE systems, ranging from pilot-scale units to large industrial extractors, coupled with the increasing consumer demand for “clean-label” and “eco-certified” products, positions this technology as a vital pillar for the biorefining of agro-industrial waste. By moving away from stagnant, high-emission extraction models to dynamic, supercritical systems, the global agri-food industry can finally bridge the gap between ecological preservation and economic viability, ensuring that the latent health benefits of vegetable waste are fully realized in the global market through the creation of a new generation of value-added ingredients that are as beneficial for the planet as they are for human health [82,83].

## 5. Thermodynamic Foundations and Multi-Scale Transport Phenomena in the Supercritical Valorization of Fruit-Processing Biorefineries

The systemic transition toward a global circular bioeconomy necessitates a fundamental re-engineering of fruit-processing by-products, specifically pomace, seeds, and peels, transforming them from voluminous environmental liabilities into high-density reservoirs of specialty lipids and secondary metabolites through the application of high-pressure supercritical fluid technology. In this framework, SFE using CO_2_ has emerged as the definitive technological paradigm for the surgical recovery of these compounds, offering a level of molecular preservation and selectivity that conventional solid–liquid extraction protocols, often hampered by the use of neurotoxic or flammable solvents like hexane, methanol, and chloroform, simply cannot replicate, as evidenced by the structural integrity and bioactivity of the resulting phyto-complexes [84,85,86]. The fundamental mechanical advantage of SFE is rooted in the tuneable density of CO_2_ beyond its critical point, where the fluid exhibits a unique hybrid of gas-like transport properties, characterized by high diffusivity and near-zero surface tension, and liquid-like solvating power that can be precisely modulated by varying the pressure and temperature of the medium to target specific molecular classes. This duality facilitates deep penetration into the complex, lignin-reinforced cell walls of fruit residues, effectively bypassing the internal mass transfer resistances and stagnant boundary layers that typically limit the efficiency of organic solvents during the leaching process, thereby enhancing the overall extraction kinetics [87,88].

### 5.1. Winery by-Products

In the specific context of winery by-products, such as grape marc and seeds (*Vitis vinifera*), the optimization of the extraction yield is inextricably linked to the manipulation of the fluid’s density, which allows for the targeted isolation of lipophilic fractions, specifically grape seed oil rich in linoleic acid and tocopherols, while maintaining a strictly anoxic environment that prevents the oxidative degradation of sensitive pi-bonds and ensures a solvent-free final product [89,90]. However, the inherent non-polarity of SC-CO_2_ presents a thermodynamic barrier for the recovery of more polar polyphenolic scaffolds; consequently, the strategic incorporation of green co-solvents (modifiers), particularly bio-ethanol or acidified hydro-alcoholic mixtures, is employed to shift the Hildebrand solubility parameters of the supercritical phase, enabling the quantitative recovery of high-molecular-weight proanthocyanidins and anthocyanins that would otherwise remain sequestered within the pomace matrix [91,92]. This process is governed by a complex interplay of internal and external mass transfer resistances, where the effective diffusion coefficient of the solute within the microporous vegetable structure becomes the rate-limiting step during the falling extraction rate period; to address this, modern biorefinery protocols increasingly utilize intensified pre-treatments such as UAE or pulsed electric fields to induce irreversible micro-poration of the vegetal tissues, thereby reducing characteristic diffusion path and maximizing the cumulative yield of targeted secoiridoids and phenolic acids [93,94,95].

### 5.2. Berry Pomace

This intensification is particularly critical for berry-derived pomace, such as that from *Rubus fruticosus* L., where the SFE cycle provides a protective barrier against the thermal stress encountered in traditional Soxhlet or reflux systems, ensuring the preservation of heat-sensitive antioxidant networks and polar lipids while simultaneously reducing the volume of waste generated [96,97,98]. Comparative studies have consistently demonstrated that SFE-derived blackberry extracts exhibit significantly higher radical scavenging potency and enzymatic inhibitory effects against lipid peroxidation than those obtained via conventional solvent leaching, a result attributed to the maintenance of the natural synergistic phyto-complex and the absence of artifacts [99,100]. At the molecular level, the solubility of these fruit-derived active agents is fundamentally governed by the density of the SC-CO_2_ which is a non-linear function of pressure and temperature as described by the Peng-Robinson equation of state, where the solvating power is further enhanced by the presence of co-solvents which induce local density fluctuations around the solute molecules, a phenomenon known as solvation clustering or micro-heterogeneity. This clustering is responsible for the dramatic increase in the solubility of relatively polar molecules like polyphenols when small amounts of ethanol are added, as it effectively lowers the threshold pressure required to achieve the transition from a solubility-limited regime to a diffusion-limited one, a parameter of paramount importance for the energy-intensive industrial processing of fruit seeds [101,102,103].

### 5.3. Citrus Residues

As we extend this logic to citrus-based residues, the precision of SFE becomes even more pronounced; for orange and mandarin peels, the technology allows for the selective fractionation of volatile terpene compounds, primarily D-limonene, from non-volatile poly-methoxylated flavones through controlled multi-stage pressure reduction (depressurization), which facilitates the collection of high-purity essential oils without the formation of thermal artifacts or off-flavours associated with steam distillation [104,105,106]. The modelling of these citrus-based systems via Response Surface Methodology has identified critical “sweet spots” where the balance between fluid density and solute vapor pressure is optimized, yielding oils with superior antibacterial and antioxidant profiles that meet the stringent requirements of the fragrance and cosmetic industries [107,108]. Furthermore, the valorization of tropical fruit residues, such as pomegranate (*Punica granatum* L.), mango (*Mangifera indica* L.), and guava, utilizes SFE to isolate punicalagins, carotenoids, and oleoresins with high oxidative stability, effectively bridging the gap between waste management and production of pharmaceutical-grade nutraceuticals [109,110,111].

### 5.4. Exotic Fruit Peels

The quantified SFE parameters applied to diverse fruit matrices are provided in Table 3, whereas Table 4 highlights the process-specific breakthroughs of SFE over conventional fruit processing methodologies. The versatility of supercritical fluids is further exemplified by the “dual-valorization” of banana peels and pear residues; while SC-CO_2_ removes high-value pigments and arbutin, the remaining solid residue undergoes structural functionalization, transforming it into a contaminant-free, high-fiber substrate suitable for the food industry, thus adhering to the “zero-waste” paradigm of modern agricultural management [112,113]. From an engineering perspective, the scalability of SFE systems, ranging from pilot-scale units to large-scale industrial extractors, is supported by rigorous mathematical descriptions of the mass transfer kinetics, where the Sovová’s broken-and-intact cell model is frequently applied to describe the transition from the constant extraction rate period to the diffusion-controlled regime, allowing for the calculation of the mass transfer coefficients (k_f_ and k_s_) which are vital for maintaining economic viability and minimizing CO_2_ consumption through optimized recycling loops [114,115]. The biological relevance of these extracts is further enhanced by the absolute absence of solvent-induced toxicity, which is particularly important for the recovery of betalains and phenolic acids from exotic fruit peels, where SFE serves as a high-throughput alternative to methanol-based leaching while maintaining the chemical fingerprint of the raw material [116,117]. The integration of subcritical water stages into the SFE workflow (Sequential SFE-SWE) has further expanded the solvating window to include highly polar glycosides, allowing for a comprehensive depletion of the biomass in a single, pressurized biorefinery cascade that recovers both lipophilic and hydrophilic fractions without cross-contamination [118,119]. This level of systemic efficiency is mirrored in the recovery of oils from passion fruit and guava seeds, where the precision of SFE prevents the geometric trans-cis isomerization of carotenoids and the polymerization of unsaturated fatty acids that typically occur during high-temperature solvent recovery [120,121,122]. By aligning ecological conservation with economic profitability, the industry can finally finalize the paradigm shift from waste disposal to strategic molecular recovery, employing hyphenated analytical techniques to validate that the extracts meet the standards of the pharmaceutical sector regarding bioavailability and chemical stability.

Ultimately, the role of supercritical fluid technology in the fruit-processing industry represents the most viable path toward a truly sustainable bioeconomy, where the molecular complexity of nature is harnessed through the thermodynamic precision of the supercritical state, ensuring that the latent health benefits of the global fruit supply chain are fully realized while minimizing the chemical footprint on the environment. This necessitates a continued focus on the non-ideal behaviour of the supercritical phase, particularly the calculation of fugacity coefficients (Φ_i_) and the use of the Fickian second law of diffusion to model the migration of solutes through the exhausted layers of the matrix, which is found to be particularly high in pomegranate seeds due to their dense lignified structure. Overcoming this resistance requires either an increase in temperature, which, however, must be balanced against the risk of thermal degradation, or the use of ultrasound-assisted SFE, where the micro-jets generated by acoustic cavitation physically erode the seed surface, creating new interfacial areas for mass transfer. This structural erosion is visible via scanning electron microscopy (SEM) and correlates directly with the shortened extraction times reported in the literature for exotic fruit residues. Furthermore, the economic modelling of these processes must account for the CO_2_ recycling efficiency; modern industrial units can recover up to 95% of the solvent, making the operating cost primarily dependent on electrical consumption for the compressors. When comparing the total cost of ownership between SFE and conventional maceration for mango peels, SFE is often found to be more expensive in the short term but significantly more profitable over a five-year horizon due to the higher market value of the solvent-free, standardized extracts. This standardized quality is essential for the cosmetic industry, which requires precise concentrations of specific terpenoids and antioxidants without the olfactory interference of organic solvent residues. As the scientific community continues to refine these high-pressure processes, the emphasis is shifting toward “smart” biorefineries where SFE is integrated with membrane separation and subcritical water hydrolysis, creating a closed-loop system that extracts, separates, and purifies bioactive molecules in a single, continuous stream, thereby optimizing the entire value chain from farm to functional ingredient.

## 6. Future Perspectives

The trajectory of supercritical fluid extraction for the next decade is defined by a shift toward industrial decarbonisation and sophisticated life cycle assessment (LCA). Modern industrial units are now reaching recovery efficiencies of over 95%, positioning SFE as a primary driver for industrial greening, especially when utilizing biogenic CO_2_. The integration of Artificial Intelligence and advanced process control represents the next frontier. Predictive algorithms and “digital twins” can analyze feedstock inconsistency in real-time, adjusting pressure and temperature to optimize solubility and minimize energy expenditure. Despite this promising outlook, several practical hurdles restrict broader industrial use. The high capital expenditure (CAPEX) and stringent high-pressure safety requirements remain significant barriers. Logistically, the seasonality of agro-industrial feedstock and the high energy costs associated with biomass drying and moisture management (which must be maintained between 5 and 12%) can impact plant throughput. Furthermore, achieving rigorous extract standardization across heterogeneous batches and navigating evolving regulatory frameworks for “zero-waste” processes are essential steps for the commercial maturation of this technology.

## 7. Conclusions

SFE represents a mature and viable pathway toward a truly circular green chemistry, supported by over three decades of industrial success in the food and pharmaceutical sectors. This review has demonstrated that by aligning thermodynamic precision with the strategic valorization of agro-industrial by-products, it is possible to recover high-purity bioactive compounds without the environmental burden of conventional solvents. While the initial investment is significant, the operational efficiency, solvent recyclability, and the production of “clean-label” ingredients justify the transition to a supercritical system. Ultimately, SFE serves as a blueprint for a sustainable future where agricultural residues are transformed from environmental waste into strategic resources, ensuring that industrial profitability and ecological stewardship are no longer in conflict.

## Figures and Tables

**Table 1:** Detailed SFE Conditions and Targeted Molecules.

Vegetable Matrix	SFE Conditions	Extracted Molecules	References
Olive leaves	250–300 bar; <50 °C, SC-CO_2_ ± bio-ethanol	Chlorophyll a and b, α-tocopherol, oleuropein	[37,46,47,48,49,50,51]
Carrot Peels	300 bar; 45 °C, pure SC-CO_2_	β-carotene, α-carotene, lutein	[59,60]
Beetroot	Pure SC-CO_2_ + polar modifiers	Betalains	[63]
Potato Peels	Pure SC-CO_2_ + polar modifiers	Chlorogenic acids	[64,65]
Turmeric Roots	Strategy: Multi-stage pressure reduction co-solvent: Coconut lipids	Curcuminoids, Volatile essential oils	[66,67]
Broccoli	low temperature pure SC-CO_2_	Sulforaphane, isothiocyanates	[68,69,70]
Tomato waste	up to 500 bar Pure SC-CO_2_	Lycopene, β-carotene	[71,72,73,74]

**Table 2:** SFE vs. Conventional Extraction Methods.

Matrix	Conventional Method	Main Limitation	SFE Advantage	References
Olive leaves	Soxhlet/Maceration	Thermal degradation High solvent use	Low T (<50 °C) Preserved antioxidants	[37,46,47,48,49,50,51]
Carrot peels	High-T reflux	Trans-to-cis isomerization	Zero isomerization Active geometry intact	[59,60]
Tomato waste	Solid–liquid leaching	High pigment oxidation Solvent residues	100% solvent-free High oxidative stability	[71,72,73,74]
Beetroot	Methanol leaching	Toxic chemical residues No “clean label”	Food-grade safety High-throughput	[63]
Broccoli	Heat-driven SLE	Sulforaphane destruction Smelly sulfur artifacts	no “off-flavours” Intact isothiocyanates	[68,69,70]
Pumpkin waste	Cold-pressing	Low diffusion Low global yields	High gas-like diffusivity Maximized yield	[75,76,77,78]

**Table 3:** Detailed SFE Conditions and Targeted Molecules for Fruit Matrices.

Fruit Matrix	Process Conditions	Recovered Target Molecules	References
Grape seeds	100–200 bar Low temperature Pure SC-CO_2_	Linoleic acid, tocopherols, grape seed oil	[84,85,121]
Grape pomace	300–500 bar Co-solvent: Ethanol or acidified hydro-alcoholic mix	Proanthocyanidins, Anthocyanins	[28,38,89]
Orange and mandarin	Pure SC-CO_2_ Controlled multi-stage depressurization	D-limonene, volatile terpenes, polymethoxylated flavones	[104,105,106]
Pomegranate seeds	Pure SC-CO_2_ No Co-solvent	Punicalagins Oleoresins	[101,102,103,109]
Berry pomace	Low thermal stress Pure SC-CO_2_	Polar lipids Antioxidant networks	[96,97,98]
Mango peels	Density-controlled fractionation	Carotenoids Standardized terpenoids	[111,117]

**Table 4:** SFE vs. Conventional Extraction Methods for Fruit Matrices.

Fruit by-Product	Conventional Method	Main Limitation	SFE Advantage	References
Grape seeds	Organic solvent SLE	Co-extracted polar impurities Lipid oxidant risk	100% pure lipophilic isolation Shielded from oxygen	[84,85,86]
Orange and mandarin	Steam distillation	Formation of thermal artifact Off-flavours in essential oils	Cold selective fractionation Native scent profile	[104,105,106]
Berry pomace	Soxhlet/reflux	Heat-sensitive network destruction High waste volumes	Unaltered fito-complex integrity High radical scavenging power	[96,97,98,99]
Pomegranate seeds	Conventional maceration	Blocked by dense lignified structures Low mass transfer	Shortened cycle time Enhanced surface erosion	[101,102,103,116]
Passion fruit	High-temperature recovery	Fatty acid polymerization Carotenoid isomerization	Prevention of geometric shifts Absolute fluid density precision	[99]
Mango peels	Classic solvent extraction	Unstable quality batch-to-batch Solvent traces remaining	Pure cosmetic-grade extracts Zero olfactory interference	[111,117,122]

## Data Availability

The original contributions presented in the study are included in the article, further inquiries can be directed to the corresponding author.
